# Rap1 signaling in endothelial barrier control

**DOI:** 10.4161/cam.27352

**Published:** 2014-01-01

**Authors:** Willem-Jan Pannekoek, Anneke Post, Johannes L Bos

**Affiliations:** Molecular Cancer Research and Cancer Genomics Netherlands; University Medical Center Utrecht; Utrecht, The Netherlands

**Keywords:** cdc42, cytoskeletal tension, endothelial barrier function, epac1, krit1, radil, rap1, rasip1, rho

## Abstract

The small G-protein Rap1 plays an important role in the regulation of endothelial barrier function, a process controlled largely by cell–cell adhesions and their connection to the actin cytoskeleton. During the various stages of barrier dynamics, different guanine nucleotide exchange factors (GEFs) control Rap1 activity, indicating that Rap1 integrates multiple input signals. Once activated, Rap1 induces numerous signaling cascades, together responsible for the increased endothelial barrier function. Most notably, Rap1 activation results in the inhibition of Rho to decrease radial stress fibers and the activation of Cdc42 to increase junctional actin. This implies that Rap regulates endothelial barrier function by dual control of cytoskeletal tension. The molecular details of the signaling pathways are becoming to be elucidated.

## Introduction

The Rap1 protein, comprising the two highly homologous isoforms Rap1A and Rap1B (95% sequence homology), is a Ras-like small G-protein that was heralded 25 y ago as an inhibitor of Ras-induced cell transformation.^1^ By now it is recognized that Rap1 has one main function: induction of cell adhesion, either to other cells or to extracellular matrix.^2^^-^^4^ Many other effects reported to occur downstream of Rap1, including inhibition of Ras-induced cell transformation, can likely be attributed to the effect of Rap1 on cell adhesion. Cell adhesion dynamics are of vital importance for many processes, including platelet coagulation, lymphocyte homing, angiogenesis, and endothelial barrier function. Genetic interference with Rap1 signaling in diverse model systems has solidly implicated Rap1 in all of these processes.^5^ Multiple regulators and effectors of Rap1 have been identified for single events, raising an important question: why are multiple activators and effectors simultaneously important to relay signaling? We are now starting to understand the conjunctive function of Rap1 regulators and effectors due to novel insights into Rap1 signaling in the regulation of the endothelial barrier function.

The endothelium is a specific layer of cells that forms the inner lining of the entire vascular system. As such, it functions as the main control system to dynamically regulate the passage of fluids, solutes, and cells through the vessel wall, a process referred to as endothelial barrier function.^6^ Regulated transport through endothelial cells (transcellular permeability) is of significant importance, although transport through intercellular cell–cell junctions (paracellular permeability) forms the most conventional mode of barrier regulation: the tighter cell–cell adhesion, the less leakage through the monolayer. Two molecular complexes regulate endothelial cell–cell adhesion: Tight Junctions (TJs) and Adherens Junctions (AJs). At TJs, the extracellular domains of transmembrane proteins, such as Claudin and Occludin of neighboring cells, interact in a homophilic manner. The cytosolic tails of these proteins interact with the zonula occludens (ZO) adaptor proteins to link to the actin cytoskeleton. The AJ is conceptually similar, although different in protein composition: VE-cadherin transmembrane proteins interact homophilically in the intercellular cleft and intracellularly bind Catenin adaptor proteins to link to the actin cytoskeleton.^7^ As such, TJs and AJs connect the actin cytoskeletons of neighboring cells, thereby conferring monolayer rigidity, but also allowing dynamic adhesion regulation, as will become evident below.

Rap1 is a small G-protein that cycles between an active, GTP-bound state and an inactive, GDP-bound state. Guanine nucleotide exchange factors (GEFs) bind Rap1 and stimulate the dissociation of GDP, thereby allowing abundant GTP to bind, activating Rap1. GTPase-activating proteins (GAPs) enhance the intrinsic GTP-hydrolyzing activity of Rap1 to inactivate the G-protein.^8^ The importance of Rap1 in controlling the endothelial barrier was recognized upon the finding that barrier tightening agents generally act by elevating the levels of cAMP, but do not solely increase barrier function by activating the cAMP-activated kinase PKA. Instead, cAMP-induced barrier tightening can be recapitulated by specifically activating the cAMP-responsive RapGEF Epac1.^9^^-^^12^ Consistently, the barrier function of endothelial monolayers in vitro can be decreased by overexpression of RapGAP^10^^,^^12^ or depletion of Rap1.^13^^-^^15^ Specific activation of Epac1-Rap1 protects isolated rat venular microvessels against platelet-activating factor (PAF)-induced permeability^16^ and in vivo Epac1-Rap1 protects against VEGF-induced dermal leakage^10^ and ventilator-induced lung injury.^17^

In terms of expression levels, Rap1B is the major Rap1 isoform in endothelial cells.^18^ However, loss of one Rap1A allele in mice severely enhances embryonic pathology of endothelial-specific Rap1B deletion (unpublished data mentioned in ref. 5). Furthermore, the barrier function of HUVEC monolayers is more sensitive to depletion of Rap1A rather than Rap1B.^13^^,^^14^ A different subcellular localization of Rap1A and Rap1B may explain this discrepancy. When overexpressed in HUVEC, Rap1A, but not Rap1B, colocalizes with VE-cadherin at cell–cell junctions and co-immunoprecipitates the junctional Rap1 effector AF6.^14^ The localization of Ras-like G-proteins depends on lipid modifications and charged residues within the C-terminal hypervariable region.^19^ Both Rap1A and Rap1B are geranylgeranylated, but subtle differences exist in their C-terminal hypervariable regions, which are likely to direct different localizations. Furthermore, differentially localized pools of Rap1 isoforms may exist. As will become evident below, Rap1 controls endothelial barrier function by regulating several downstream pathways. It is conceivable that these pathways are controlled by differentially localized pools of Rap1, although data to support this theory are lacking at the moment.

## Endothelial Barrier is Controlled by Multiple RapGEFs

The endothelial monolayer is subjected to permeability-inducing agents and permeability-decreasing agents and it will dynamically adjust its barrier function based on the relative presence of these stimuli.^20^ Rap1 activity is important during different stages of permeability dynamics (Fig. 1): (1) Endothelial monolayers maintain a certain baseline, steady-state level of permeability (also referred to as normal permeability),^21^ which we will hereafter refer to as “basal barrier function.” Basal barrier function is decreased in cells depleted of Rap1.^13^^,^^14^ (2) Hyperpermeability can be induced by growth factors or agents like VEGF or thrombin upon request of the underlying tissue. Once the need for hyperpermeability declines, barrier is restored to basal, which we will call “barrier recovery.” In monolayers depleted of Rap1, barrier recovery is prolonged.^22^ (3) Lastly, high levels of barrier can be achieved by cAMP-inducing agents, which we will refer to as “barrier tightening.” Absolute barrier levels upon tightening are decreased when Rap1 is depleted.^13^

**Figure:**
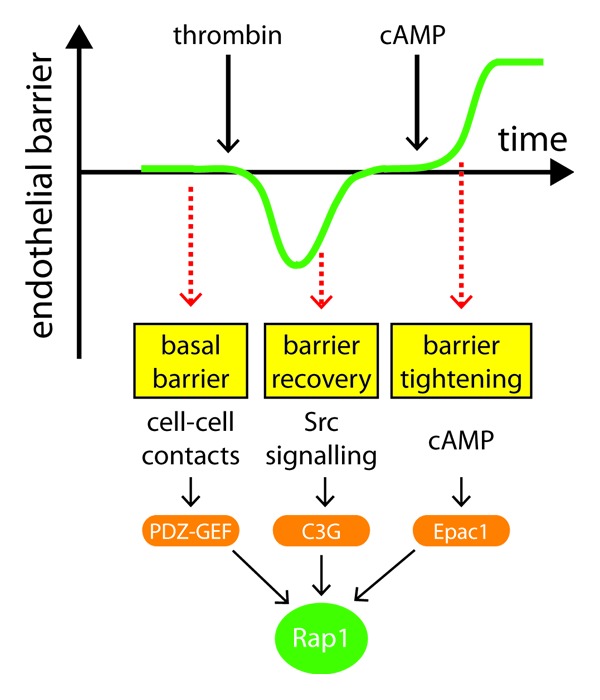
**Figure 1.** Schematic representation of RapGEF function during different stages of barrier control. At steady-state, endothelial monolayers confer a certain level of barrier function, termed basal barrier function, during which Rap1 is continuously activated by PDZ-GEFs, possibly downstream of VE-cadherin engagement at cell–cell contacts. After induction of hyperpermeability, for instance by thrombin, barrier function is actively restored to basal levels, termed barrier recovery, during which Rap1 is activated by Src-activated C3G. High levels of barrier function (barrier tightening) are induced by cAMP via activation of the RapGEF Epac1.

It can be hypothesized that barrier levels during these stages are regulated by the same permeability-promoting and antagonizing signaling pathways, the relative strengths of which will determine absolute barrier levels.^21^ However, it appears that there is a differential requirement of the RapGEFs C3G, PDZ-GEF, and Epac1 during different stages of permeability based on the hierarchical effects they have on barrier regulation (Fig. 1): C3G is not required for basal barrier function (our unpublished results) but is implicated in barrier recovery after hyperpermeability.^22^ In contrast, depletion of PDZ-GEF does affect basal barrier function.^13^ Activation of Epac1 induces high levels of barrier tightening and desensitizes monolayers to PDZ-GEF depletion.^13^ However, one could imagine that when barrier measurements are performed under conditions with low PDZ-GEF activity and abundant growth factors (cAMP present) some Epac1-dependency of basal barrier function could be measured. Indeed, an Epac1-dependent effect on basal barrier function of HUVEC has been reported.^23^ In vivo, similar differences in PDZ-GEF-responsiveness and cAMP-induction might exist between different endothelial cells along the vascular tree. Epac1 deletion in mice is claimed to enhance basal permeability in skin, muscle, and fat tissue,^24^ although it remains to be determined to what extent this phenotype reflects basal barrier function rather than failure to respond to cAMP-inducing agents. Therefore, it is important to stress that our definition of basal barrier function is a state of intrinsic, Rap1-dependent, barrier regulation when cAMP levels are low.

### Basal barrier function: PDZ-GEF

Motivated by the finding that basal barrier function required Rap1 activity but not the cAMP-regulated endothelial RapGEF Epac1, the PDZ-GEFs were identified to control Rap1 activity under basal conditions.^13^ PDZ-GEF1 and PDZ-GEF2 are two RapGEFs that are highly similar in domain architecture and which are both implicated in the regulation of epithelial cell–cell junctions.^3^ In the control of endothelial barrier function, PDZ-GEF1 is the main isoform.^13^ PDZ-GEF1 deletion causes embryonic lethality as a result of defective yolk sac vasculogenesis,^25^^,^^26^ which can be attributed to defective cell–cell adhesion.^27^ Given the permeabilizing effects of PDZ-GEF1 depletion or Rap1 depletion under basal conditions, we envision that PDZ-GEF1-Rap1 functions to prevent excessive barrier leakage during steady-state, probably in response to a stimulus that is constitutively present. Importantly, this stimulus is not cAMP. Although PDZ-GEF1 resembles Epac1 in the sense that it bears a cyclic nucleotide-binding domain, in vitro activation of Rap1 by PDZ-GEF1 cannot be induced by cyclic nucleotides.^28^^,^^29^ Alternatively, the presence of cell–cell contacts could confer constitutive PDZ-GEF1 activation. Rap1 is activated at cell–cell contacts upon engagement of VE-cadherin in a manner dependent on MAGI-1, which is a junctional scaffold protein that binds β-catenin and PDZ-GEF1.^30^ It remains unclear whether MAGI-1 binding is sufficient for PDZ-GEF1 activation or whether it is merely required for PDZ-GEF1 activation. In case of the latter, other junctional proteins could be participating. VE-cadherin engagement activates the GTPase Rac1,^31^ which stabilizes the endothelial barrier through its effector cortactin.^32^ Interestingly, cortactin depleted HUVEC monolayers display decreased basal levels of Rap1 activity. This coincides with increased permeability under basal conditions, but not upon activation of Epac1-Rap1, perfectly mimicking the effect of PDZ-GEF depletion.^33^ Clearly, the molecular details on PDZ-GEF1 activation await further research.

### Barrier recovery: C3G

Upon request of the underlying tissue, the endothelial barrier can be decreased to allow passage of solutes or cells. Thrombin and VEGF are examples of such permeabilizing factors. Restoration of basal barrier function after hyperpermeability is not just dependent on these factors diminishing, but can be actively induced.^22^ Thrombin induces activation of the key permeability-inducing protein Rho. Following the Rho-induced permeability increase, Rap1 becomes activated to restore basal barrier function. Indeed, inhibition of Rap1 signaling does prolong the duration of barrier recovery.^22^ Barrier recovery is similarly affected by inhibition of Src signaling. Interestingly, Rap1 activation during barrier recovery coincides with enhanced Src phosphorylation and increased Src-mediated phosphorylation of C3G,^22^ which is known to relieve C3G autoinhibition.^34^ These results were obtained using thrombin to challenge the monolayer, but C3G-mediated activation of Rap1 has also been suggested upon the permeability-inducing proteins VEGF and TIMP-2.^35^^-^^37^ Technically, the effect of C3G depletion on barrier recovery remains to be shown, although it is encouraging that C3G is similarly implicated in barrier recovery of epithelial monolayers: Rap1 becomes activated when an epithelial monolayer is challenged to restore the monolayer, which correlates with C3G binding to E-cadherin.^38^^,^^39^ Here, dominant negative C3G does prevent AJ reassembly after monolayer challenge.^39^

### Barrier tightening: Epac1

When vascular leakage exceeds the need of the underlying tissues, additional tightening of the endothelial monolayer can be induced by agents like adrenomedullin and prostaglandin E2.^40^^,^^41^ These vasoactive agents function by increasing intracellular levels of cAMP. The main cAMP targets are PKA and the Epac proteins, which function in concert to tighten the endothelial barrier.^42^^,^^43^ Epac is of particular importance here, as its key function is to activate Rap signaling upon binding of cAMP.^44^^-^^46^ The generation of an Epac-selective analog of cAMP (named 007) boosted the identification of processes induced by Epac activation, among which is endothelial barrier tightening. Actually, the aforementioned reports that implicated Rap1 to be involved in endothelial barrier function all made use of 007, thereby simultaneously implicating Epac1 in endothelial barrier function. Hence, Epac1-Rap1 induces tightening in cultured monolayers and isolated rat venules and in vivo protects against VEGF-induced dermal leakage and ventilator-induced lung injury.^9^^-^^12^^,^^16^^,^^17^ Epac exists as two isoforms: Epac1 and Epac2, but only Epac1 is expressed in endothelial cells, which is in agreement with barrier tightening being sensitive to Epac1 depletion and the vascular leakage defects observed in Epac1−/− mice.^11^^,^^23^^,^^24^^,^^47^ It should be noted that although Epac1 activates Rap1, not all Epac1 effects can be attributed to the activation of Rap1: whereas depletion of Epac1 completely abolishes the effect of 007 on barrier tightening, depletion of Rap1 only has a partial effect.^13^ This cannot be simply explained by inefficient Rap1 knockdown, as depletion of the downstream pathway Rasip1-Radil-ArhGAP29 (see below) completely prevents barrier tightening induced by an active mutant of Rap1, but simultaneously allows a similar partial increase by 007.^48^ This cAMP-induced, Rap1-independent barrier tightening effect of Epac1 is mediated by microtubules (MTs).^23^ How Epac1 regulates MTs is still elusive. Interestingly, cAMP induces Epac1 translocation independent of its effect on Epac1 auto-inhibition.^49^ As such, Epac1 could function as a cAMP-regulated adaptor protein to recruit binding partners, such as AKAP9. AKAP9 is required for Epac1-induced MT growth. In addition, AKAP9 is required for adhesion of HUVEC on surfaces coated with the Fibronectin peptide RGD, but not on Fc-VE-cadherin-coated surfaces, suggesting Epac1-MT functions to activate integrins.^50^ These activated integrins do not induce barrier tightening by enhancing cell-matrix interactions, as integrin-mediated adhesion to matrix is dispensable for 007-induced barrier tightening.^43^ Instead, these integrins are suggested to function at cell-cell contacts.^50^ Indeed, 007 induces αv and α5 integrin relocation to cell-cell contacts, where they are required for 007-induced barrier tightening.^50^

## Rap1 Releases Cytoskeletal Tension

Rap1 tightens the endothelial barrier in a manner dependent of AJs^11^^,^^13^ and induces adhesion of HUVEC on Fc-VE-cadherin-coated surfaces.^10^^,^^51^ Using immunofluorescence, the intensity of VE-cadherin staining at cell–cell contacts seems increased upon Rap1 activation,^9^^,^^10^^,^^12^ although this is not accompanied by increased VE-cadherin staining using flow cytometry,^43^ suggesting that Rap1 does not function to increase VE-cadherin transport to the plasma membrane, but rather changes the morphology or dynamics of cell junctions to make VE-cadherin staining appear more intense. This is supported by FRAP analyses on VE-cadherin, which indicate that the lateral mobility of VE-cadherin in cell–cell junctions is impaired by 007.^52^ Junction morphology and dynamics are tightly controlled by the actin cytoskeleton.^53^ Indeed, Rap1-induced barrier tightening is accompanied by gross changes in the actin cytoskeleton: the amount and intensity of radial stress fibers (RSFs) decrease, whereas increased amounts of F-actin colocalize with VE-cadherin (referred to as “junctional actin”). These Rap1-induced actin changes are independent of AJ presence,^11^ whereas the Rap1-induced lateral mobility decrease of VE-cadherin in cell–cell junctions and Rap1-mediated barrier tightening are sensitive to actin-depolymerizing agents.^23^^,^^52^ Hence, Rap1 regulates the actin cytoskeleton to control endothelial barrier function.

The actin cytoskeleton dynamically regulates the AJ by applying a physical force on the junction.^53^ When permeability is induced, RSFs localize in bundles perpendicular to the cell-cell contacts and confer high tension on the junction, causing the AJs to appear discontinuous (also referred to as punctuate, zipper-like, or focal AJs).^53^^,^^54^ When cytoskeletal tension declines, AJs will become linear and actin fibers will run parallel to or even colocalize with the cell–cell contact.^53^ Cytoskeletal tension is conferred by the sliding of Myosin motors along the actin filaments.^55^ To enable sliding, regulatory Myosin Light Chain 2 (MLC2) needs to be phosphorylated on its T18 and S19 residues, which can be induced by the kinase ROCK.^56^ ROCK in turn is activated by the Rho proteins. As such, the activity of Rho can be considered the key gatekeeper of cytoskeletal tension.

It has been suggested in a multitude of cellular contexts that Rap1 functions to control Rho-mediated cytoskeletal tension in both positive and negative ways.^57^^-^^61^ In the endothelium, the situation seems clear-cut: Rap1-depleted monolayers display clear RSFs and discontinuous AJs (indicative of high radial tension), whereas monolayers stimulated with 007 are largely devoid of RSFs, but rather have junctional actin and linear AJs (low radial tension).^13^^,^^42^ This has initiated the search to how Rap1 inhibits Rho signaling. Currently, two different molecular mechanisms have been identified.

### Tension reduction by KRIT1

KRIT1 is a FERM domain-containing adaptor protein that was first identified as a Rap1 interacting protein,^62^ and subsequently shown to act downstream of Rap1 in endothelial barrier function.^63^ The molecular mechanism is largely elucidated: KRIT1 binds to MTs, but when active Rap1 binds the FERM domain, KRIT1 translocates to AJs, where it binds β-catenin.^63^^-^^65^ In accordance, a KRIT1 mutant protein with decreased affinity for Rap1 displays increased MT binding and decreased junction localization, the latter being rescued by MT disruption.^66^ This same mutant is unable to rescue the decreased endothelial barrier function upon KRIT1 depletion in vitro and fails to rescue the dilated heart phenotype found in KRIT1-morphant zebrafish in vivo. KRIT1 inhibits Rho activity, which was revealed by mice with heterozygous loss of KRIT1 or the KRIT1 interacting protein CCM2. These mice display increased vascular permeability, which coincides with increased levels of phosphorylated MLC2.^66^^-^^68^ In both mice, the vascular leakage defect can be completely restored by treatment with fasudil, an inhibitor of the Rho effector ROCK.^67^ In humans, mutations in KRIT1 and CCM2 are known to cause cerebral cavernous malformations (CCMs), which are vascular malformations in the central nervous system characterized by dilated vessels that are prone to leakage.^69^^,^^70^ Although KRIT1+/− mice show increased vascular permeability, these mice lack the typical malformations observed in the human disease. However, these lesions can be induced by simultaneously compromising DNA mismatch repair, suggesting a two-hit model for the human disease.^71^ Importantly, the prevalence of these CCM lesions can be decreased by fasudil.^72^

It is unclear how KRIT1 and CCM2 control Rho signaling. KRIT1 deletion was shown to change Rho activity but not Rho protein levels.^67^ In contrast, depletion of KRIT1 or CCM2 in cultured endothelial cells does not only increase the amount of active Rho, but also increases overall Rho protein levels.^68^^,^^73^ KRIT1 depletion causes nuclear translocation of its binding partner β-catenin to increase β-catenin-mediated transcription.^74^ In addition, KRIT1 itself can also translocate to the nucleus.^75^ Hence, KRIT1 may control endothelial barrier function via either direct control of Rho activity or by transcriptionally controlling Rho proteins or even Rho regulators. Clearly, both of these putative mechanisms need further exploration.

### Tension reduction by Rasip1

Rasip1 is a recently characterized effector of Rap1,^48^^,^^76^ the expression of which co-clusters with endothelial-specific genes.^77^ During development, Rasip1 is essential for blood vessel formation, as shown using Rasip1−/− mice and morpholino’s in *Xenopus*.^76^^,^^78^^,^^79^ In human, a SNP in the Rasip1 locus is associated with retinal venular caliber,^80^ indicating the importance of Rasip1 for endothelial function. Rasip1 binds the RhoGAP ArhGAP29 to regulate Rho and the downstream phosphorylation of MLC2, which likely underlies the defective blood vessel formation in animal models, as impaired lumen formation of Rasip1-depleted or ArhGAP29-depleted HUVEC in matrigel can be rescued by dominant-negative RhoA.^79^ Concomitantly, HUVEC monolayers depleted of Rasip1 show discontinuous AJs and increased RSFs, resulting in decreased basal barrier function.^76^ Thus, Rasip1 depletion is sufficient to affect basal barrier function of HUVEC, but this phenotype is enhanced by simultaneous depletion of the Rasip1 homolog Radil,^48^ which has also been shown to bind ArhGAP29.^81^ The effects of Radil/Rasip1 depletion on barrier function and RSFs are phenocopied by depletion of ArhGAP29^48^ and impairment of basal barrier function by ArhGAP29 depletion is relieved by simultaneous depletion of Rho, suggesting that Rap1 functions by reducing Rho activity through Rasip1 and ArhGAP29.^48^ In line with this, increased Rho-GTP levels are observed upon depletion of Rasip1 or ArhGAP29,^79^ although others could not find changes in Rho activity upon Rasip1 depletion^76^ or Rap1 activation.^48^ Further research on this issue is required.

Rasip1 was originally claimed to be an effector of Ras, although less efficient binding to Rap1 was also observed.^82^ However, its depletion phenotype on HUVEC strikingly resembles Rap1 depletion, suggesting Rasip1 to be regulated by Rap1. Indeed, activation-dependent binding of Rap1 to Rasip1 can be convincingly shown using GST-pull-down or FRET assays.^48^^,^^76^ Furthermore, Rap1 signaling is required for junctional localization of Rasip1.^76^ Hence, the Rasip1/Radil-ArhGAP29-Rho module constitutes a signaling cascade by which Rap1 reduces radial tension to increase endothelial barrier function.

## Rap1 Increases Junctional Tension

Activation of Rap1 dissolves RSFs and induces linear junction cables to tighten the barrier. Importantly, this effect is not entirely phenocopied by inhibition of radial tension: tension inhibitors cause RSFs to disappear and junctional actin increase, but this junctional actin is not organized in straight, linear cables, but rather takes a curved, winding appearance (Fig. 2A and B, middle panels).^13^^,^^83^^,^^84^ This winding actin phenotype coincides with decreased endothelial barrier function, as opposed to the increased endothelial barrier function when junctional actin appears straight.^13^ This suggests that while barrier tightening requires radial tension to be released, some tension should be present within the junction for proper barrier function (Fig. 2A). In this case, the direction of the tension runs along the cell–cell contact rather than perpendicular to it, which is very similar to force generation at the zonula adherens in fully polarized epithelia.^53^ We call this “junctional tension,” which was recently shown to be regulated by Rap1: while Rap1 relieves radial tension by inhibiting the Rho/ROCK pathway, resulting in decreased levels of phosphorylated MLC2 on T18 and S19, Rap1 simultaneously activates Cdc42 and its effector MRCK, which induces monophosphorylation of MLC2 on S19 (Fig. 2B). Whereas phospho-T18-S19-MLC2 appears at RSFs, phospho-S19-MLC2 appears at cell–cell contacts, suggesting monophosphorylation of MLC2 regulates junctional tension.^83^ Hence, Rap1 exerts a dual regulation of tension: radial tension on the AJs is decreased, whereas junctional tension is increased (Fig. 2A). The latter effect requires Cdc42 and MRCK, although it has to be noted that the induction of phospho-S19-MLC2 at cell–cell contacts by 007 is still partially sensitive to inhibition of Rho and ROCK.^83^ Indeed, in immortalized mouse endothelial cells, 007 induces diphosphorylated MLC2 at cell–cell contacts in a manner dependent on Raf-1 and ROCK2.^85^ Considering the differences in MLC2 phosphorylation status at cell–cell contacts in these reports, further research is required on junctional tension and the underlying signaling mechanisms, but the novel concept of dual tension regulation by Rap1 offers a very intriguing new view on Rap1 function.

**Figure:**
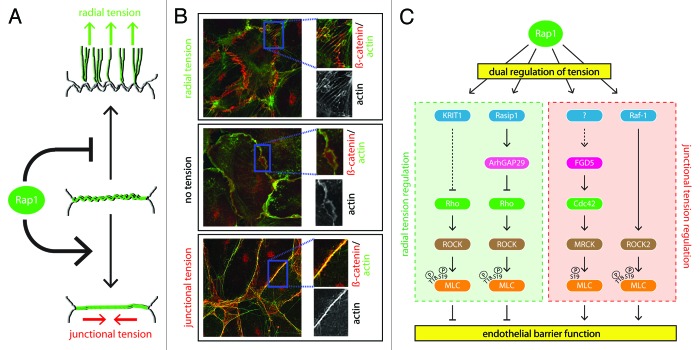
**Figure 2.** Dual regulation of tension by Rap1. (**A**) Rap1 regulates endothelial barrier by a two-fold regulation of cytoskeletal tension. First, Rap1 relieves tension on radial stress fibers, thereby decreasing the radial tension on the junction (upper cartoon). Second, Rap1 induces tension in linear actin cables along the cell–cell contact (junctional tension, lower cartoon). The middle cartoon is a normally non-existent, intermediate state (winding actin) when no tension is present, which can be induced by inhibiting Myosin. Cell–cell contacts are shown in gray, actin cables are shown in green. (**B**) Immunofluorescence pictures showing the different actin morphologies depicted in 2A, which were induced by ArhGAP29 depletion (upper box), blebbistatin treatment (100 µM, 30 min) (middle box), or 007 treatment (1 µM, 10 min) (lower box). Actin in shown in green, whereas the AJ marker β-catenin is shown in red. (**C**) Molecular mechanisms of tension regulation employed by Rap1 to regulate endothelial barrier function, comprising both regulation of radial tension (left box, green) and regulation of junctional tension (right box, red). See text for details.

## Additional or Secondary Effects of Rap1 Activation

The dual-tension-regulation mechanism that Rap1 exerts on the actin cytoskeleton would be sufficient to explain the effects Rap1 has on the lateral mobility of VE-cadherin and endothelial barrier function (Fig. 2). However, additional effects and effectors have been suggested. For instance, oxidized phospholipids induce barrier tightening by creating cross-interactions between AJs and TJs in a Rap1-dependent manner.^86^ These Rap1-induced AJ-TJ interactions depend on the Rap1 effector AF6, which is also the suggested Rap1 effector in barrier recovery.^22^^,^^87^ Intriguingly, AF6 depletion causes sustained levels of phosphorylated MLC2 during barrier recovery.^22^ Hence, AF6 might also fulfill a role in the dual-tension mechanism. Another important role in Rap1-mediated barrier regulation may be fulfilled by Rac. Barrier tightening by prostaglandin E2, prostacyclin and ANP coincides with Rap1-dependent activation of Rac and Rac is also activated upon Epac1 induction with 007.^17^^,^^42^^,^^88^ Rac activity is of vital importance for endothelial barrier function, as both dominant-negative and constitutively active mutants increase HUVEC permeability.^89^ Its function in endothelial junctions is multifaceted: cadherin trafficking, stabilization of the cadherin-catenin complex, and polymerization of junctional actin are controlled by Rac.^90^ Furthermore, dynamic interplay exists between Rac and Rho signaling,^91^ suggesting that Rac could add an additional layer of control to the dual tension mechanism. It remains to be determined which of these Rac effects on barrier function occur under the control of Rap1, and whether this occurs directly or indirectly.

## Concluding Remarks

Rap1 is a master regulator in endothelial barrier function. Multiple activation signals, mediated by various RapGEFs, confer the activation of Rap1, which then activates a variety of signaling cascades to induce endothelial barrier function. Here, we have discussed how these activators and effectors function together. Clearly, several RapGEFs are involved in different aspects of endothelial barrier regulation (Fig. 1). Here, the molecular mechanisms of Epac1 activation are well understood, but little is known on how and where PDZ-GEF and C3G are activated. Also, additional levels of Rap1 regulation may exist. For instance, RapGAPs may be involved in barrier regulation,^92^ but how they contribute to endothelial barrier function is unclear. A critical event in the regulation of endothelial barrier by Rap1 is the control of cytoskeletal tension. Importantly, Rap1 exerts a dual control on tension: whereas radial tension is decreased by Rap1, tension in the junction is induced (Fig. 2). The molecular mechanism through which Rap1 differentially regulates tensions is partially elucidated, but many questions remain. For instance, what other Rap1 effectors are required for barrier function and how do these pathways interact with each other? Do different Rap1-effector pools exist in space or in time? And what is the function of Rap2 proteins in endothelial barrier function? They have a Rap1-opposing effect,^92^ the mechanism of which remains to be determined. Is this effect also achieved by modulating tension?

Hyperpermeability of endothelial monolayers results in edema formation during thermal injury, acute lung injury, and reperfusion injury. Furthermore, hyperpermeability underlies the microvascular defects in Diabetes Mellitus.^93^ Epac1 is a dominant activator of Rap1, which potently increases endothelial barrier function. As such, Epac1-Rap1 offers a very suitable therapeutic target to treat aforementioned pathologies. WS^®^ 1442 is a standardized, safe proven extract from the *Crataegus* hawthorn, which in vitro tightens endothelial barrier by activating Epac1-Rap1, and in vivo prevents inflammation-induced hyperpermeability in the mouse cremaster muscle,^94^ paving a road to target Epac1-Rap1 in the treatment of hyperpermeability. 007 seems to be the most promising way to achieve this: 007 specifically activates Epac1-Rap1,^95^ significantly limiting putative side effects that would result from general cAMP inducers. Furthermore, 007 induces superactivation of Epac1, meaning that the maximal activity of Epac1 obtainable with saturating concentrations of 007 is much higher than the maximal activity of Epac1 stimulated with cAMP.^96^ Hence, if you are to treat hyperpermeability, 007 seems the way to go. How to get 007 in your endothelium? The ball is up to the pharmacists!

## Notes

10.4161/cam.27352
